# mHealth Adoption by the Older Adults: A Structured Literature Review

**DOI:** 10.12688/f1000research.172707.1

**Published:** 2025-12-04

**Authors:** Raghavendra G, Shashidhara Y N, Poornima Panduranga Kundapur, Jyothi Mallya

**Affiliations:** 1 Computer Science, Welcomgroup Graduate School of Hotel Administration, Manipal Academy of Higher Education, Manipal, Udupi, Karnataka, 576104, India; 2 Department of Community Health Nursing, Manipal College of Nursing, Manipal, Manipal Academy of Higher Education, Manipal, Udupi, Karnataka, 576104, India; 3 Computer Science, Manipal Institute of Technology, Manipal Academy of Higher Education., Manipal, Udupi, Karnataka, 576104, India; 4 Library, Welcomgroup Graduate School of Hotel Administration, Manipal Academy of Higher Education, Manipal, Udupi, Karnatka, 576104, India

**Keywords:** mHealth, SDG3, SDG10, good health and well-being, reduced inequalities, technological innovation, older adults, structured review

## Abstract

**Background:**

A structured review on mHealth adoption among older adults is needed understand and synthesize existing evidence on the factors influencing their engagement with various mHealth technologies. Such a review helps policymakers, healthcare providers, and technology designers in developing age-friendly mobile health interventions that enhance older adults’ well-being and healthcare access. Thus, this review aims to synthesise and provide a detailed summary of older adults’ mHealth engagement and adoption literature from a business perspective.

**Methods:**

This study adopts the systematic procedure and rationale for the systematic literature review (SPAR-4-SLR) protocol to achieve the study’s objective. Nineteen articles from the Scopus and Web of Science databases related to mHealth engagement and adoption by older adults are included in this study.

**Results:**

The findings revealed that the majority of the studies are atheoretical. The Unified Theory of Acceptance and Use of Technology is used most in this domain. The review findings also reveal a complex interplay of technological, psychological, and social factors influencing adoption. It has also been found that quantitative methods have frequently been used to examine the adoption of mHealth among older adults.

**Conclusion:**

The study concludes with a constructive discussion of the existing theoretical, methodological, and contextual gaps in mHealth engagement and adoption literature.

## Introduction

The global demographic landscape is shifting profoundly, with the population of older adults growing unprecedentedly. This growing trend can be attributed to increased life expectancy and declining fertility rates ( Rahman, 2019). According to the World Health Organization (2023), by 2050, one in six people will be 60 years or older ( World Health Organisation, 2023). This demographic transformation highlights the urgency of addressing the aging population’s health and well-being needs. One promising solution lies in the growing domain of mobile health (mHealth) apps, a subset of digital health technologies that leverages mobile devices, applications, and wireless infrastructure to support healthcare delivery, monitoring, and self-management.

Studies found that engagement with mHealth technologies among older adults is influenced by various factors. For example, it is found that family health positively correlates intending to use mHealth devices among older adults ( Chang et al., 2024). Perceived independence, understanding of technology, and visibility of benefits are key facilitators ( Kruse et al., 2017). The study findings also suggest that trust in healthcare providers and strong health self-efficacy positively influence adoption ( Schroeder et al., 2024). Regarding the usability, findings revealed that older adults can efficiently use mHealth interventions like wearable activity trackers and health apps, indicating high satisfaction and acceptability ( Reyes et al., 2025).

Despite increasing scholarly attention to smart homes ( Forestal & Li, 2022; Marikyan et al., 2019), IoT systems ( Perez et al., 2023), and healthcare robotics ( Dankar & Badr, 2022) for elderly care, limited research addresses how older adults engage with mobile health (mHealth) technologies from a business research perspective. Most existing studies emphasize technical applications or healthcare outcomes, neglecting user engagement’s theoretical, contextual, and methodological dimensions. In response to these theoretical and methodological gaps, this study conducts a structured literature review (SLR) using the TCM (Theory–Context–Methodology) framework to comprehensively analyze and synthesize prior research on elderly engagement with mHealth technologies. The TCM framework allows for a multidimensional evaluation, identifying what has been studied and highlighting *how*, *where*, and *under what assumptions* these studies have been conducted. Thus, this review intends to address the following research questions:

RQ1: What are the theories and models of mHealth adoption existing among older adults?

RQ2: How is the knowledge adoption of mHealth among older adults currently obtained?

RQ3: Where should future research on adopting mHealth by older adults be heading?

This review aims to advance scholarly understanding and offer actionable insights for researchers, practitioners, and policymakers involved in designing age-inclusive digital health ecosystems by systematically mapping the current state of knowledge and uncovering key research gaps. In doing so, it contributes to both the aging and digital health literatures and sets the stage for future investigations that are more theoretically robust, contextually sensitive, and methodologically diverse.

## Research methodology

### Review protocol

**Figure 1.**
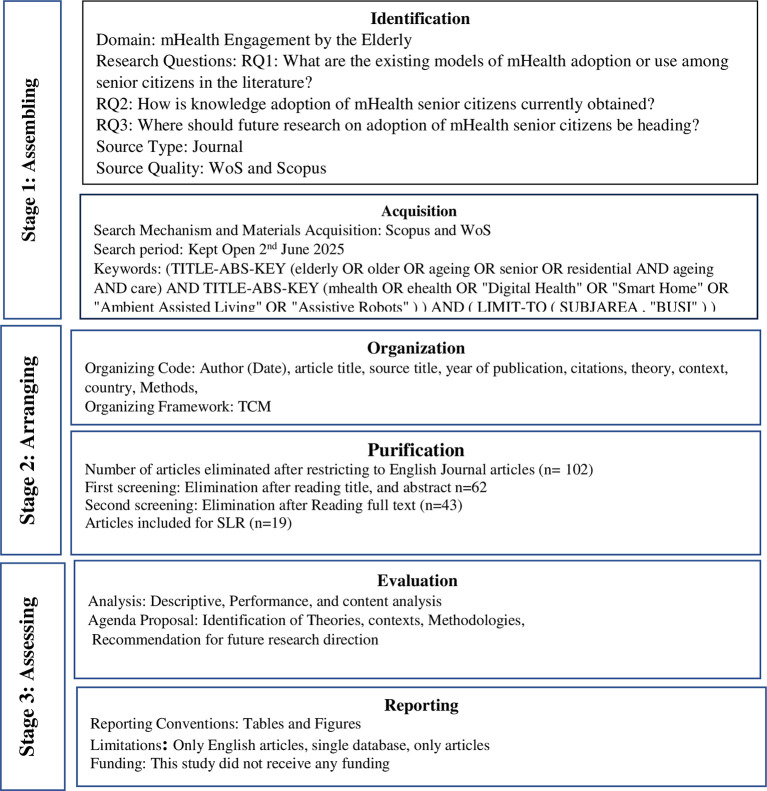
The SPAR Model used for SLR. This figure illustrates three stages of the SPAR Model. They are assembling, arranging, and assessing. The model also outlines the sub-stages, such as identification, acquisition, organization, purification, evaluation, and reporting.

The Scientific Procedure and Rationale for Systematic Literature Review (SPAR-4-SLR) protocol is used in this study to address key limitations in existing systematic review guidelines, such as PRISMA and PRISMA-P ( Moher et al., 2016). While these frameworks support transparent and structured reporting, they often lack detailed justifications for methodological choices ( Paul et al., 2021). SPAR-4-SLR fills this gap by providing a step-by-step process that outlines what researchers should do during an SLR and explains why each step is necessary. This systematic process enhances the review process’s rigor, clarity, and academic defensibility, making SPAR-4-SLR especially valuable for researchers seeking methodological precision and theoretical grounding in their review design. The SPAR-4-SLR comprises of three stages and six sub-stages that flow sequentially ( Figure 1).

*Stage 1: Assembling, involving sub-stages identification and acquisition*

Identification

The identification substage includes the study area, research questions, source type, and source quality. The area or topic of this SLR is mHealth engagement by older adults. It explores mHealth engagement among older adults by analysing peer-reviewed journal articles indexed in WoS and Scopus. The study addresses three key research questions: (RQ1) What existing models explain mHealth adoption or use among senior citizens? (RQ2) How is knowledge regarding elderly adoption of mHealth currently obtained? (RQ3) What directions should future research on mHealth adoption in senior populations pursue?

Acquisition

The literature search was conducted in Scopus and Web of Science, with the search period open until 2nd June 2025. The search key string used was: (TITLE-ABS-KEY (elderly OR older OR ageing OR senior OR residential AND ageing AND care) AND TITLE-ABS-KEY (mhealth OR ehealth OR “Digital Health” OR “Smart Home” OR “Ambient Assisted Living” OR “Assistive Robots”)) AND (LIMIT-TO (SUBJAREA, “BUSI”)), ensuring a focus on digital health and elderly care within the business context.

*Stage 2: Arranging, comprising sub-stages: organisation and purification*

Organisation

In the organisation sub-stage, all selected articles were systematically coded using key bibliographic and analytical elements: Author (Date), article title, source title, year of publication, citations, theory, context, country, and methods. To ensure analytical consistency and thematic alignment, the TCM (Theory-Context-Method) framework is used ( Paul et al., 2023). This framework facilitated a structured synthesis of findings, allowing the review to identify theoretical foundations, methodological approaches, and contextual relevance in mHealth adoption among older adults.

Purification

In the purification sub-stage, a systematic screening process was undertaken to ensure the relevance and quality of the selected articles. Initially, 102 articles were retained after restricting the search to English-language journal publications. The first screening, based on title and abstract review, led to the elimination of 62 articles. A second screening, involving full-text reading, further excluded 43 articles. Ultimately, 19 articles met all inclusion criteria and were deemed suitable for detailed analysis in the systematic literature review.

*Stage 3: Arranging*

Arranging, consisting of sub-stages: evaluation and reporting.

Evaluation

The selected articles underwent descriptive, performance, and content analysis in the evaluation sub-stage. This process facilitated the identification of key theories, research contexts, and methodologies used in mHealth adoption among older adults. Based on these insights, an agenda was proposed with recommendations for future research directions to bridge identified gaps.

Reporting

In the reporting sub-stage, structured tables and figures are employed to present key findings, themes, and classifications clearly and concisely.

## Results

To address the first research question— “What are the existing theories or models of mHealth adoption or use among senior citizens in the literature?”—we identified and synthesized key theoretical frameworks applied in mHealth adoption by the older adults. Our study findings revealed that diverse theories and models offering different perspectives were adopted. We have discussed them in detail in the following paragraph.

### Theories and models

The analysis reveals a lack of theoretical grounding in most studies, with 63% not using any formal framework. UTAUT was the most cited model ( Frishammar, Essén, Simms, et al., 2023b; Leung et al., 2024), indicating its relevance in mHealth adoption. This finding highlights the need for more theory-driven research to enhance consistency and depth in future investigations ( Table 1).

**Table 1.** Theories adopted by the mHealth researchers.

No.	Theory/models	Explanation	Citations
1	PMT	PMT is a psychological theory that explains how individuals are motivated to adopt protective behaviours when faced with threats to their health or well-being ( Rogers 1975).	( Chiu, Won, and Chen 2025)
2	IISM	ISSM, also known as the DeLone and McLean IS Success Model, is a widely accepted framework for evaluating the effectiveness and impact of information systems ( DeLone and McLean 2002).	( Oderanti et al. 2021)
3	UTAUT	UTAUT explains that individuals’ adoption of a technology and subsequent usage behavior are based on four key constructs: performance expectancy, effort expectancy, social influence, and facilitating conditions ( Venkatesh et al. 2003).	( Frishammar, Essén, Simms, et al. 2023b; Leung et al. 2024)
4	VAM	VAM is a theoretical framework used to explain why users adopt new technologies based on their perceived value ( Kim, Chan, and Gupta 2007).	( Xiong and Zuo 2022)
5	IRT	IRT was proposed by Ram and Sheth (1989) to explain why consumers resist adopting innovations, even when those innovations are beneficial. Unlike models like UTAUT or TAM, which focus on factors promoting technology adoption, IRT centres on the barriers and psychological resistance that prevent or delay adoption.	( Leung et al. 2024)

PMT = Protection Motivation Theory, ISSM = Information Systems Success Model, UTAUT = Unified theory of acceptance and use of technology, VAM = Value-Based Adoption Model, IRT = Innovation Resistance Theory.

This table provides the details of the different theories used in mHealth researchers, along with the citations.

Unified theory of acceptance and use of technology (UTAUT)

UTAUT assumes that an individuals’ behavioral intention to use technology is influenced by performance expectancy (perceived usefulness), effort expectancy (ease of use), social influence (peer and societal pressure), and facilitating conditions (availability of resources) ( Venkatesh et al., 2003). In the context of mHealth adoption by older adults literature, the UTAUT model was used to study the Digital health platforms (DHP) ( Frishammar, Essén, Bergström, et al., 2023a). The findings of the study revealed that older adults perceive a few barriers to initial adoption of DHPs, such as technology anxiety and trust.

Innovation Resistance Theory (IRT)

IRT ( Ram and Sheth, 1989) identifies the factors influencing individuals’ resistance to adopting any innovations, even when such innovations may offer clear advantages. Their study identifies five barriers, namely usage, value, risk, tradition, and image barriers. This theory was applied to understand older adults’ barriers to adopting mHealth applications. Leung et al. (2024) explicitly investigate perceived complexity and dispositional resistance to change in adopting the mHealth app. The findings of their study reveal that technology anxiety negatively impacted adoption intention. On the other hand, the perceived usefulness of the mHealth app positively influenced adoption.

Protection Motivation Theory (PMT)

PMT is a psychological theory originally used in health communication ( Rogers, 1975) that explains how individuals are motivated to protect themselves from threats or harmful situations. Since its inception, this theory has also been applied in technology adoption, such as cybersecurity ( Shah Alam et al., 2024), health behaviour ( Balla & Hagger, 2025), and AI services ( Park et al., 2024). In the context of older adults, Chiu et al. (2025) applied PMT to examine older adults’ adoption of mHealth apps. The study found that coping appraisals, particularly response efficacy, were stronger predictors of adoption intention than threat appraisals. Findings also revealed that technology readiness significantly influenced these appraisals, highlighting the role of optimism and discomfort in shaping perceptions.

Synthesis of research objectives and key findings

The following section summarizes selected articles included in the study to provide a current research landscape on mHealth adoption by older adults (Annexure I). The primary research objectives and corresponding findings are consolidated to identify common patterns, gaps, and emerging directions in the field, offering a foundation for future inquiry and practical application in designing age-inclusive mHealth solutions.

The literature reveals a complex interplay of technological, psychological, and social factors influencing adoption. For example, perceived usefulness emerged as one of the key themes of mHealth adoption. Leung et al. (2024) developed a framework for Health Task Management Support (HTMS) and found that features supporting medical, dietary, and exercise tasks significantly enhance perceived usefulness. However, barriers, such as perceived complexity and resistance to change, contribute to technology anxiety, negatively impacting adoption intentions. The age-related differences in digital behavior are also prominent. Frishammar, Essén, Simms, et al. (2023b) observed that older adults use digital healthcare platforms differently than younger users, often spending more time navigating them. This outcome aligns with findings from Frishammar, Essén, Bergström, et al. (2023a), who identified negative attitudes and technology anxiety as major barriers for elderly users. Their integrated framework suggests that addressing these psychological barriers is essential for improving adoption. The design and accessibility of digital platforms are also found to be critical. Bosilj Vuksic et al. (2023) highlighted that the lack of digital literacy, unequal access, and platform complexity hinder adoption among older adults. Similarly, Yan et al. (2023) emphasized the importance of sensory-friendly and emotionally supportive interfaces, recommending multi-sensory and simplified designs to enhance usability.

From a behavioral perspective, Chiu et al. (2025) found that coping appraisals—especially response efficacy—strongly influence older adults’ attitudes toward mHealth. Their study highlights the role of technology readiness and coping mechanisms in shaping adoption intentions, suggesting that non-linear, multifactorial relationships govern user behavior.

The role of social support is another recurring theme. Tipaldi & Natter (2022) showed that family members often act as co-decision makers, and framing decisions around age-related needs can boost confidence in older adults. Lie et al. (2016) further emphasized the importance of trust-based relationships with caregivers and institutions, suggesting that trust can mitigate the disruption caused by new technologies. Privacy concerns also emerge as a significant barrier. Lee et al. (2019) demonstrated how smart home systems could expose elders to privacy risks through sensor data analysis, raising ethical concerns about surveillance and data security.

Additionally, the broader socio-economic and policy context plays a role. Leporatti & Montefiori (2024) found that poor digital skills correlate with worsened health outcomes, especially in countries with widespread eHealth systems. Their findings advocate for stronger digital literacy programs and inclusive eHealth policies. While mHealth technologies offer significant potential to enhance elderly care, their adoption is contingent on addressing usability, psychological readiness, social support, and systemic barriers. Future interventions must be holistic, user-centered, and policy-informed to ensure equitable and effective integration of mHealth into aging populations.

Our second research question was - *How is knowledge adoption of mHealth older adult currently obtained?* To achieve this objective, the context and methodologies adopted by the researchers in the mHealth adoption among older adults was synthesized, analysed and discussed below.

### Context

The reviewed studies highlight a growing intersection of aging populations and digital health technologies, particularly in developing, adopting, and impacting various digital platforms and smart technologies to support elderly care. For instance, many studies focus on mHealth apps and digital health platforms, addressing behavioral and systemic barriers to adoption. Leung et al. (2024) and Chiu et al. (2025), for example, investigate the adoption of mHealth applications among older adults, incorporating psychological constructs like innovation resistance and protection motivation theory. Similarly, Frishammar et al. (2023) explore motivations and post-adoption behaviors related to digital healthcare platforms, identifying key challenges older adults face.

Few studies have also investigated smart home technologies as facilitators of aging. Mao et al. (2024), Yan et al. (2023), and Tipaldi and Natter (2022) examine how smart homes enhance life satisfaction and influence decision-making among older adults. Issues like interface design and privacy concerns are also highlighted ( Ehrenhard et al., 2014; Lee et al., 2019). Other than smart homes, technologies like telepresence robots ( Schulz et al., 2025) and social robots ( Khaksar et al., 2016) are investigated for their roles in facilitating remote consultations and reducing social vulnerability. Broader system-level studies address eHealth and mobile platforms ( Oderanti et al., 2021; Xiong & Zuo, 2022), digital communication ( Sanders et al., 2015), and digital skills and policy implications ( Leporatti & Montefiori, 2024), reflecting how digital inclusion and literacy remain central to elderly engagement.

Methodology

Among studies examining older adults’ adoption of mobile health (mHealth) apps and digital health technologies, quantitative methods are the most commonly adopted approach ( Chiu et al., 2025; Mao et al., 2024; Tipaldi & Natter, 2022; Xiong & Zuo, 2022). Researchers employed survey-based quantitative designs to explore factors influencing adoption behavior, perceived usefulness, and technology readiness. Combining qualitative and quantitative data, mixed-method approaches are also widely used ( Frishammar, Essén, Bergström, et al., 2023a; Leung et al., 2024), offering a richer understanding of motivations and barriers. In addition, qualitative studies ( Bosilj Vuksic et al., 2023; Lie et al., 2016) provide in-depth insights into older adults’ trust, experiences, and perspectives on digital health. Experimental ( Lee et al., 2019; Schulz et al., 2025), user-centered design ( Yan et al., 2023), and participatory action research ( Compagna & Kohlbacher, 2015) methods are less common but contribute valuable design- and interaction-oriented perspectives to this growing field.

Data collection tool

Among the studies exploring older adults’ adoption of mHealth apps, questionnaires emerged as the most commonly used data collection tools ( Chiu et al., 2025; Leporatti & Montefiori, 2024; Tipaldi & Natter, 2022; Xiong & Zuo, 2022; Yan et al., 2023). Questionnaires enable researchers to gather large-scale, structured data on perceptions, attitudes, and behaviors. The second most used data collection tool is interviews and focus group discussions ( Frishammar, Essén, Bergström, et al., 2023a; Leung et al., 2024; Sanders et al., 2015). These tools help researchers gain deeper insights into participants’ experiences and motivations. Other data sources were secondary sources, such as reports ( Mao et al., 2024) and participatory methods ( Lie et al., 2016), while others combined multiple tools ( Khaksar et al., 2016), reflecting a diverse methodological landscape in this field.

Our third research question was Where *should future research on adopting mHealth by older adults be heading?* To address this research question, we have recommended future research questions based on research gaps in theoretical, methodological, and contextual perspectives.

## Future research directions

### Theoretical perspectives

While many studies have employed established theories, such as UTAUT, IRT, and PMT, the theoretical application remains sparse and uneven. Several studies fail to ground their investigations in formal theory, limiting their explanatory power and generalizability. Therefore, it is recommended that future research should adopt a more theory-driven approach by integrating behavioral, sociological, and psychological theories/frameworks to capture older adults’ mHealth app adoption holistically. Some theoretical lenses, like Socio-Emotional Selectivity Theory and Digital Divide Theory, could deepen understanding of intrinsic motivations and structural barriers, which are discussed below.

Socio-Emotional Selectivity Theory (SEST)

SEST posits that people prioritize emotionally meaningful goals and experiences over knowledge acquisition as they age. This theory helps explain why older adults may prefer mHealth technologies that offer emotional support or social connectivity ( Carstensen, 2021).

SEST, developed by Laura Carstensen (2021), posits that as individuals age, their perception of time becomes more limited, leading them to prioritize emotionally meaningful goals and relationships over knowledge acquisition or novelty. This shift in motivational focus has profound implications for how older adults interact with technology, including mHealth applications. Older adults are more likely to engage with mHealth technologies that support their emotional well-being. For example, apps that facilitate communication with caregivers or provide reassuring health feedback may be more appealing than those focused solely on data tracking. SEST suggests that emotional resonance is a key driver of engagement, meaning that technologies perceived as emotionally supportive are more likely to be adopted and used consistently. Therefore, future researchers can use this theory to understand how older adults’ shifting emotional priorities influence their engagement with mHealth technologies.

Digital divide theory

The Digital Divide Theory (DDT) refers to the gap in terms of access to, use of, or knowledge of information and communication technologies (ICT) ( van Dijk, 2013). This digital divide is classified into three categories: First level is access to digital technologies, second level is digital skills and usage patterns, and third level is outcomes or benefits derived from the ICT ( van Deursen & van Dijk, 2014). The digital divide is critical in the context of mHealth adoption by older adults. For example, older adults often face several types of digital exclusions, such as limited smartphone access, high-speed internet, low digital literacy, and technology anxiety ( Choi & Dinitto, 2013). These factors likely hinder their engagement and subsequent adoption of mHealth apps effectively despite the benefits they offer.

Applying DDT to mHealth adoption offers a deeper understanding of why older adults lag in utilizing these digital health services. For instance, first-level divide issues (access to mHealth app or technology) may include affordability of devices or infrastructure in rural areas. Similarly, the second-level challenges include navigating app interfaces or interpreting health information. Third-level divides become evident when older adults do not achieve the expected health outcomes from mHealth usage due to suboptimal engagement or misinterpretation of data ( Robinson et al., 2015). Recognising these gaps is essential for designing mHealth interventions. Therefore, future research in the mHealth adoption should focus on these three levels of digital divide to create an age-friendly interface design and supportive social environment.

### Methodological perspectives

The review findings revealed that most studies rely on qualitative interviews, surveys, or mixed methods methodologically, with relatively few adopting experimental, participatory, or user-centered design approaches. While surveys provide valuable insights at scale, they are often limited in capturing the lived experiences of older adults. Future research should expand the use of participatory action research (PAR) and scenario-based design, particularly in designing inclusive smart technologies and healthcare platforms. Additionally, integrating longitudinal tracking or digital trace data (e.g., usage logs from mHealth apps or smart devices) could offer more accurate behavioral insights over time. Multi-modal research methods—blending qualitative input with real-time data collection and AI-driven analysis—can yield a richer understanding of adoption processes and drop-off points. There is also a strong need for cross-national comparative research using harmonized data sets to examine how different policy landscapes and levels of digital infrastructure impact older adults’ engagement with technology. Finally, underrepresented populations, such as rural elderly, low-income seniors, or those with disabilities, should be more purposefully included to ensure findings are inclusive and equitable.

### Contextual perspectives

Future research on mHealth adoption among older adults should consider diversifying the contextual settings to capture the complexity of usage environments and user needs better. While current studies primarily examine mHealth apps in general ( Chiu et al., 2025; Leung et al., 2024), future studies could focus on specific health conditions, such as chronic disease management, mental health support, or post-hospitalization care, to understand contextual differences in technology acceptance and sustained use. Moreover, as digital health platforms become increasingly integrated into healthcare systems ( Frishammar, Essén, Simms, et al., 2023b), research should explore the interoperability between mHealth apps and institutional healthcare databases, addressing concerns around privacy, data-sharing, and real-time clinical decision support. Additionally, there is a need to explore mHealth adoption in cross-cultural and rural versus urban contexts, especially considering differences in infrastructure, digital literacy, and cultural attitudes toward technology and aging. For instance, Xiong and Zuo (2022) discussed the relevance of contextual factors in China’s mobile medical platforms. Similar context-specific studies across low- and middle-income countries would help expand the global understanding of mHealth equity.

The intersection of mHealth and smart environments, such as smart homes or wearable monitoring systems ( Lie et al., 2016; Mao et al., 2024), also opens avenues for research on ecosystem-level integration. Therefore, future studies could examine how older adults navigate between multiple technologies, such as apps, sensors, and voice interfaces, and how such convergence affects their trust, usage patterns, and health outcomes. Finally, research should delve into policy and institutional factors, such as digital health literacy programs, reimbursement models, and regulatory frameworks ( Leporatti & Montefiori, 2024), to understand systemic enablers and barriers. Such multidimensional and context-rich explorations will help design inclusive, sustainable, and scalable mHealth solutions tailored to the diverse needs of older adults.

### Limitations

While this review offers a structured and transparent method for synthesizing existing research, they have limitations. Some key limitations are as follows. There may be source bias since this review is based on the two databases. Therefore, it is recommended that future studies should include articles from the regional journals, grey literature or non-indexed sources. While structured literature reviews effectively map what has been studied, they may fall short in critically analysing why findings occur or how concepts interrelate, especially if the review lacks a strong theoretical or conceptual framework. A theoretical lens enables a deeper interpretation of patterns and contradictions in the literature. SLRs are typically one-time snapshots of a field and do not adapt to new research trends unless regularly updated. As a result, they may quickly become outdated in dynamic disciplines. Therefore, it is recommended that future studies should focus on meta-analysis since the findings are generalizable. Given that this review is within the broad business research domain, future review studies should delve deeper into the intersection of technology diffusion, user engagement, and digital service innovation about older adult populations.

## Conclusion

This study aimed to explore the complex landscape of mHealth engagement among older adults using a structured literature review framework- Theory-Context-Methodology framework. Situated within the business research domain, this review integrates insights from healthcare and gerontechnology to synthesize fragmented knowledge and identify key theories, models, contexts, gaps, and opportunities in the existing literature. Our analysis reveals that while mHealth technologies hold significant promise for enhancing elderly well-being, engagement remains uneven due to cognitive, emotional, technological, and contextual barriers. Most existing studies are grounded in individual-level theories such as the TAM, PMT, and UTAUT, often overlooking the broader organizational, cultural, and policy-related dimensions. Furthermore, a notable bias exists toward quantitative methods, with limited use of mixed-method or longitudinal approaches that could better capture the evolving and experiential nature of elderly engagement with technology. From a contextual standpoint, much of the research remains concentrated in high-income countries, underrepresenting the voices and experiences of elderly populations in low- and middle-income regions. Moreover, the business implications of mHealth adoption, such as service innovation, market segmentation, and digital inclusivity, remain underexplored in mainstream business research. This review also identifies several future research directions. First, there is a need for cross-cultural and intergenerational studies, including emotion-driven and trust-based models, and the integration of design thinking or co-creation frameworks to enhance user-centric development. Researchers are also encouraged to adopt more holistic, participatory, and adaptive methodologies to capture the lived experiences of elderly users over time. It can be concluded that mHealth engagement among older adults is a multidimensional phenomenon that requires interdisciplinary approaches and contextual sensitivity. By mapping the existing research through the TCM lens, this study not only clarifies the field’s current state but also lays the foundation for more impactful, inclusive, and business-relevant investigations in the future.

## Declaration of generative AI

In preparing this manuscript, GAI and AI-assisted technology (ChatGPT) were used solely to enhance readability and clarity of language. All AI-generated content was carefully reviewed and edited to ensure accuracy and integrity. The authors take full responsibility for the content and affirm that AI was not used for data analysis or generating research insights. Any potential limitations of AI-generated text were addressed through human verification.

## Data Availability

Data are available under the terms of the Creative Commons Attribution 4.0 International license (CC-BY 4.0) at https://data.mendeley.com/datasets/6t4knpsgdx/1 Citation: G, Raghavendra; YN, Shashidhara; Panduranga Kundapur, Poornima; Mallya, Jyothi (2025), “mHealth and older adults”, Mendeley Data, V2, doi: 10.17632/6t4knpsgdx.2
