# Supplementary material for: mHealth Adoption by the Older Adults: A Structured Literature Review
Source: F1000Res. 2025 Dec 4;14:1358. [Version 1] doi: 10.12688/f1000research.172707.1 (PMC12756596; doi:10.12688/f1000research.172707.1)
Supplement: Peer review history

# Reviewer report: Reviewer response for version 1

**DOI:** 10.5256/f1000research.190453.r442242

**Published:** 2026-01-06

**Authors:** Aruditya Jasrotia

**Affiliations:** 1 Amity Institute of Travel and Tourism Amity University, Noida, UP, India

The study presents a clear and relevant focus on mHealth adoption among older adults, highlighting an important gap in understanding engagement from a business perspective, which is a notable strength. The background effectively justifies the need for a structured review and clearly articulates the practical relevance for policymakers, healthcare providers, and technology designers. The use of the SPAR-4-SLR protocol demonstrates methodological rigor, and the selection of Scopus and Web of Science enhances the credibility of the review. However, the study could be strengthened by briefly clarifying the criteria used to narrow the literature to nineteen studies and by more explicitly linking the “business perspective” to the findings and implications. The results section offers useful insights into theoretical and methodological trends but would benefit from slightly more specificity regarding key adoption factors identified. Additionally, the conclusion could be enhanced by more clearly emphasizing the practical and theoretical contributions of identifying these gaps, particularly in relation to SDG3 and SDG10. Overall, the chapter is well-structured and informative, and with minor refinements in clarity and emphasis, it can more strongly convey the significance and contribution of the study.

Is the review written in accessible language?

Yes

Are all factual statements correct and adequately supported by citations?

Yes

Are the conclusions drawn appropriate in the context of the current research literature?

Yes

Is the topic of the review discussed comprehensively in the context of the current literature?

Yes

Reviewer Expertise:

NA

I confirm that I have read this submission and believe that I have an appropriate level of expertise to confirm that it is of an acceptable scientific standard.

# Reviewer report: Reviewer response for version 1

**DOI:** 10.5256/f1000research.190453.r442241

**Published:** 2025-12-31

**Authors:** Bindi Varghese

**Affiliations:** 1 CHRIST (Deemed to be University), Bengaluru, Karnataka, India

 The study presents a well-structured and comprehensive review of existing literature on mHealth adoption among older adults. The methodological rigor adopted in the literature selection and analysis process is appropriate and clearly articulated. The manuscript effectively synthesizes prior research, identifies key determinants influencing adoption, and highlights existing research gaps, thereby offering valuable insights for researchers, practitioners, and policymakers in the field of digital health and gerontology.

 The manuscript’s clarity of presentation, coherence of arguments, and relevance to contemporary healthcare challenges.

Is the review written in accessible language?

Yes

Are all factual statements correct and adequately supported by citations?

Yes

Are the conclusions drawn appropriate in the context of the current research literature?

Yes

Is the topic of the review discussed comprehensively in the context of the current literature?

Yes

Reviewer Expertise:

Marketing, branding and leisure

I confirm that I have read this submission and believe that I have an appropriate level of expertise to confirm that it is of an acceptable scientific standard.

# Reviewer report: Reviewer response for version 1

**DOI:** 10.5256/f1000research.190453.r442239

**Published:** 2025-12-31

**Authors:** Jeetesh Kumar

**Affiliations:** 1 Taylor's University, Subang Jaya, Malaysia

- The paper addresses a highly relevant and timely topic, and the focus on older adults’ mHealth adoption from a business research perspective is commendable; however, the notion of a “business perspective” could be made more explicit by linking findings more clearly to service design, value creation, and digital health markets.
- The use of the SPAR-4-SLR protocol strengthens methodological rigour. Yet, the authors could improve transparency by briefly justifying why only Scopus and Web of Science were selected and how this choice may shape the scope of evidence.
- The research questions are clear and logically sequenced, but RQ2 on “knowledge adoption” would benefit from sharper conceptual clarity, as it sometimes overlaps with discussions of adoption and usage.
- Applying the TCM framework is a strong analytical choice; however, the synthesis within each TCM dimension could be more integrative by explicitly comparing patterns across theories, contexts, and methods rather than listing them sequentially.
- The finding that most studies are atheoretical is essential. However, the paper could strengthen its contribution by critically discussing why theory adoption remains weak in this domain and what this implies for cumulative knowledge building.
- The discussion of UTAUT, IRT, and PMT is well articulated, but some theoretical sections lean toward description; a deeper critical comparison of their explanatory strengths and limitations for older adults would enhance analytical depth.
- The results section offers rich thematic insights, yet including only 19 studies raises concerns about coverage; acknowledging how this small corpus affects generalizability would improve balance.
- Contextual analysis is a strength of the paper, particularly the inclusion of smart homes and robotics; however, more precise differentiation between mHealth and adjacent digital health technologies would reduce conceptual blurring.
- The methodological review is comprehensive, but the paper could go further by identifying concrete methodological blind spots (e.g., the lack of longitudinal or experimental designs) and providing more substantial justification.
- The future research agenda is detailed and thoughtful. However, it may benefit from prioritisation by highlighting the most urgent or high-impact directions rather than presenting all suggestions with equal weight.

Is the review written in accessible language?

Partly

Are all factual statements correct and adequately supported by citations?

Yes

Are the conclusions drawn appropriate in the context of the current research literature?

Partly

Is the topic of the review discussed comprehensively in the context of the current literature?

Yes

Reviewer Expertise:

Health tourism

I confirm that I have read this submission and believe that I have an appropriate level of expertise to confirm that it is of an acceptable scientific standard.
